# Experimental research and analysis on the resistance characteristics of simulated ore bin in water

**DOI:** 10.1038/s41598-022-17287-9

**Published:** 2022-08-02

**Authors:** Linjing Xiao, Qiang Liu, Weimin Huang

**Affiliations:** College of Mechanical & Electronic Engineering, Shandong University of Science and Technology, Qingdao, 266590 China

**Keywords:** Computational science, Software, Mechanical engineering

## Abstract

In order to research the variation law of the longitudinal resistance coefficient of the ore bin in the marine mining system under different length–diameter ratio, external shape, additional weight and Reynolds number, a set of experimental system for testing the resistance coefficient was designed and built independently. By analyzing the experimental results, it can be seen that under the same conditions, the resistance coefficient decreases gradually with the increase of Reynolds number and finally fluctuates around a certain value. Increasing the excitation displacement will reduce the overall resistance coefficient of the ore bin. The smaller the length-diameter ratio is, the larger the corresponding force value when the vibration acceleration of the ore bin is 0, and the larger the overall resistance coefficient is. The resistance coefficient of the cylindrical section is greater than that of the rectangular shape. In order to reduce the longitudinal vibration and the transverse towing offset, the shape of the ore bin should be cylindrical in actual design and production. At low Reynolds number, the increase of added weight will increase the resistance coefficient, while at high Reynolds number, the change of added weight will not cause the change of resistance coefficient.

## Introduction

**Figure 1:**
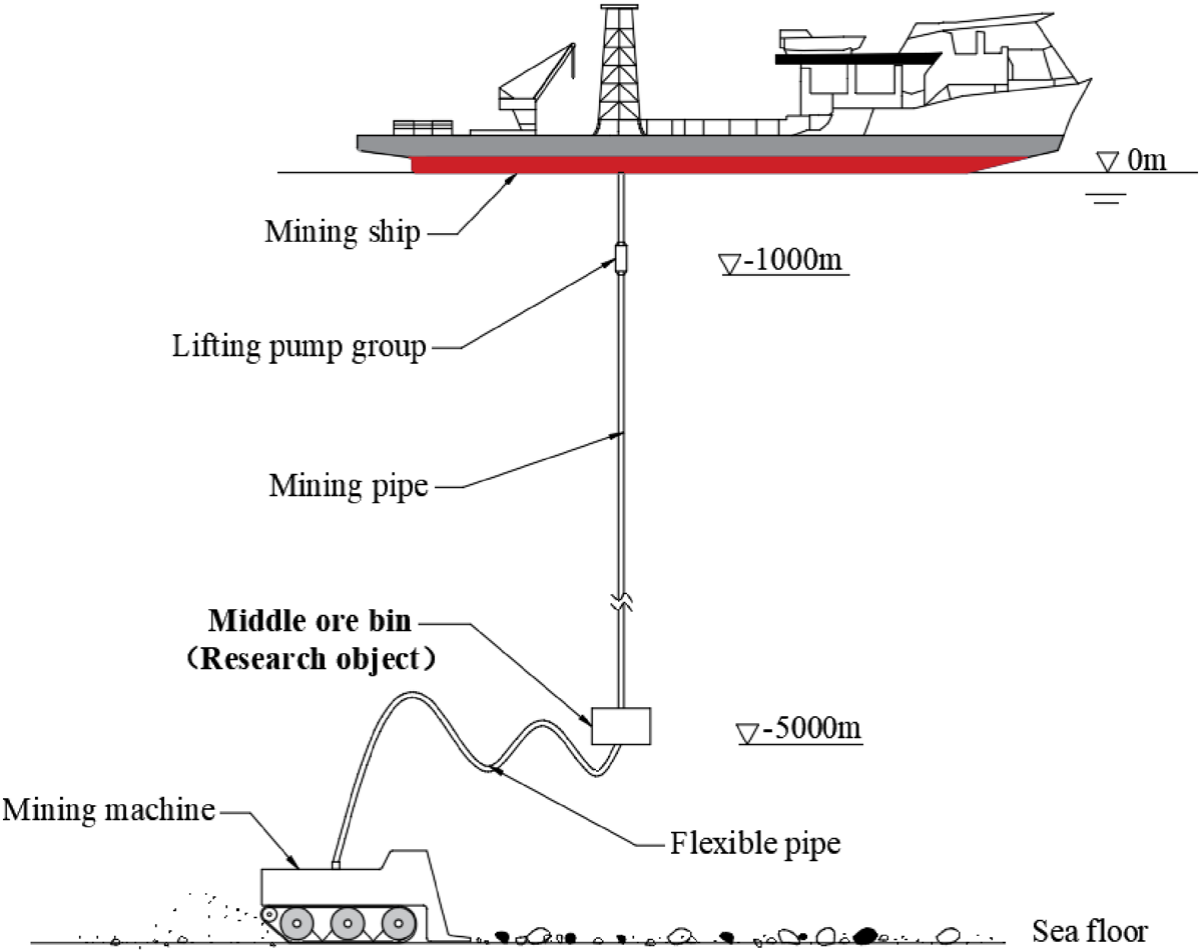
Schematic diagram of deep sea mining system structure.

In recent years, with the increasing attention and investment of various countries in the ocean development, the marine engineering industry has developed vigorously^1–3^. The seabed contains solid mineral resources with high quality and abundant resources. These mineral resources are rich in nickel, cobalt, copper, manganese and other metals, which are the core elements of aerospace, special alloys, lithium batteries, superconductors and fuel cells^4–6^. The rapid economic development has intensified the consumption of land resources, and the mineral resources under the sea have also become an important strategic target for all countries in the world^7–9^. Mining of seabed resources requires the use of deep sea mining system, as shown in Fig. 1, which is mainly composed of mining ship, mining pipe, intermediate ore bin (cylindrical) and mining locomotive^10^. As an important part of the mining system, the load force generated in the longitudinal direction of the intermediate ore bin can directly affect the longitudinal vibration and deformation of the ore mining pipe^11–13^. The key to solve the axial load force of ore bin is to determine the appropriate resistance coefficient of ore bin, and the resistance coefficient of ore bin with different shape is also different. Therefore, in order to ensure the safety and stability of mining system, the research on the resistance coefficient of ore bin has important theoretical significance and engineering application value.

Igerichick studied the cylinder model with smooth surface, proposed the correlation between the Reynolds number and the resistance coefficient, and presented the change curve of the drag coefficient with the Reynolds number^14^. Kazuo Aso studied the variation rule of the resistance coefficient and additional mass coefficient of the simulated ore bin in water by setting up the vibration test bench. When the *KC* number increases, the resistance coefficient decreases exponentially, while the additional mass coefficient decreases linearly^15,16^. *KC* number has always been considered as a parameter describing the periodic characteristics of waves, Li Yucheng made an experimental study on the force exerted on vertical square columns under wave action, and believed that the resistance coefficient of the column was a function of *KC* number, Reynolds number, model roughness and time, and gave the change of the resistance coefficient with the phase angle^17–20^. Ali Vakil used CFD software to study the relationship between drag coefficient and slenderness ratio when flowing around cylinders with different slenderness ratios at low Reynolds numbers^21^. Mao Jun used large eddy simulation and Reynolds number averaging to conduct numerical simulation and analysis of the flow resistance characteristics around a finite-length cylinder at high Reynolds number, obtained the variation law of the cylinder resistance coefficient with the aspect ratio and Reynolds number, and discussed the influence of the end face effect on the resistance coefficient^22^. Guo Xiaohui used the Lattice Boltzmann method to analyze the relationship between the resistance coefficient and Reynolds number of a cylinder with a small slenderness ratio in the range of *Re* = 0.1 ~ 100^23^. Xing Zhizhuang analyzed the horizontal pipe close to the seabed and calculated the resistance coefficient of the horizontal pipe by using the second-order Stocks wave theory and the least square weighted average method^24^. Gui Fukun studied the influence of wave elements on the resistance coefficient of horizontally fixed cylindrical rod through experiments, the increase of wave height would cause the resistance coefficient to decay exponentially, and gave an empirical formula for the resistance coefficient^25^. Liu Guijie studied the resistance coefficient of cylindrical platform pile leg through simulation experimental. Under the condition of low Reynolds number, the resistance coefficient of the model increases with the increase of Reynolds number, and the increment trend gradually slows down^26,27^. (The simulation experiment here refers to the experimental scene simulated by the author in his own experimental center according to the actual working conditions in the literature.)

In summary, there are more studies on the flow resistance coefficient around cylindrical objects at the present stage, for example, reference^28^ carried out numerical simulation on the characteristics of flow resistance around a finite-length cylinder, and obtained the variation law of the cylinder resistance coefficient with aspect ratio and Reynolds number. Reference^29^ studied the flow characteristics around a cylinder in different arrangements, spacing ratios and diameter ratios through numerical simulation. Reference^30^ based on the high-precision spectral element method, a single cylinder was taken as the research object to conduct direct numerical simulation of the flow field under the sub-critical Reynolds number, and discussed the characteristics of turbulent wake of parallel cylinders and the changing mechanism of hydrodynamic characteristics of cylinders under turbulent flow. Reference^31^ based on the viscous flow theory, a numerical wave flume model was established by using the finite volume method, and the wave force on the cylinder was numerically calculated. The improved Morison equation was proposed, which could take into account the condition that the cylinder was completely exposed to air, and the hydrodynamic coefficient was obtained through a large number of data fitting. But there is a lack of research on the variation law of the longitudinal resistance coefficient of the cylindrical ore bin under forced vibration. In this paper, a set of test system to test the longitudinal resistance coefficient is set up, and the simulation of the ore bin is studied. The variation law of the resistance coefficient under different length-diameter ratio, shape and additional mass is analyzed, and the resistance coefficient value of the ore bin under different working conditions is determined, which provides useful reference for the vibration reduction and overall design of the marine mining system.

## Theoretical analysis

The following assumptions are made before theoretical analysis:

1. Water is an incompressible non-viscous non-rotational fluid, and the system is in a stable state without external excitation;
2. When there is an excitation effect, the influence of the wall effect is ignored;
3. When the exciter drives the ore bin to move, the influence of the wave generated by the excitation on the ore bin is ignored.

When the forced vibration is applied to a cylinder in still water, the resistance coefficient can be calculated by the following formula^32,33^:

1 $$ C_{d} = \frac{2F}{{\rho Av^{2} }} $$

where, $F$ is the force change of simulated ore bin in seawater under forced vibration; $\rho$ is the fluid density; $A$ is the inflow area of the ore bin; $v$ is the relative velocity.

The Reynolds number definition formula can be expressed as^34^:

2 $$ Re{ = }\frac{v}{\upsilon }D $$

where, $D$ is the characteristic length of the measured body; $\upsilon$ is the kinematic viscosity coefficient of the fluid, and $\upsilon = 1.0067 \times 10^{ - 6} {\text{m}}^{2} /s$^35^.

## Experimental model

**Figure 2:**
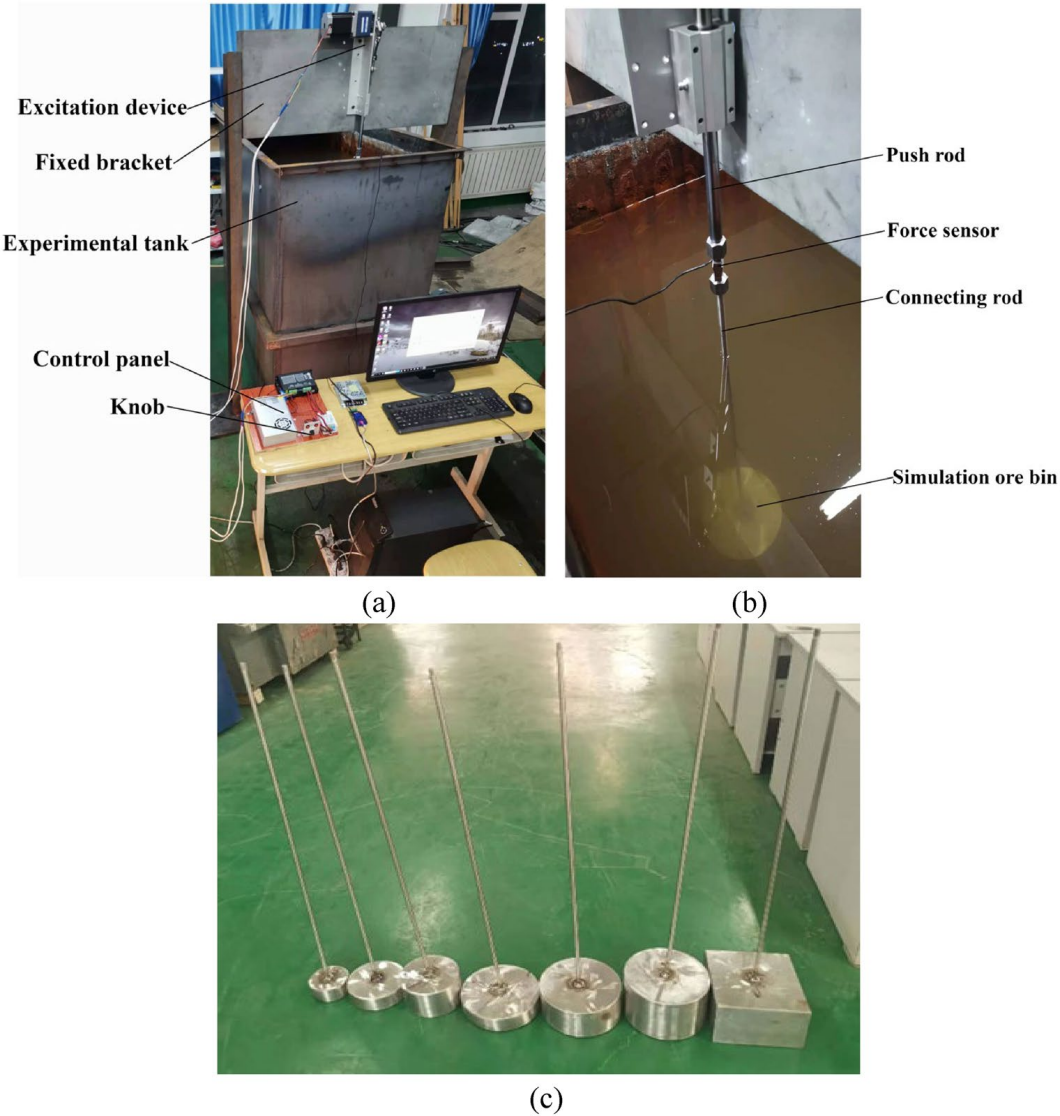
Composition diagram of experimental test system.

In order to better measure the resistance coefficient of simulated ore bin, a set of experimental system was built, as shown in Fig. 2. The size of the experimental tank is 2.5 m × 1.2 m × 1.8 m (length × width × depth), and the experimental system is mainly composed of excitation device, force sensor and the measured object (ore bin). The push rod of the excitation device is connected with the upper end of the force sensor, and the lower end of the force sensor is connected with the connecting rod of the simulation ore bin, the lower end of the connecting rod is fixed to the simulation ore bin of various shapes. The acceleration sensor is installed at the bottom of the ore bin, and the push rod of the excitation device drives the simulation ore bin to make vertical movement in the water. The vibration device is installed on the fixed bracket, which is higher than the height of the water tank. The speed of the push rod movement can be adjusted in real time through the knob of the control panel, and the displacement of the push rod can also be set by itself. In this experimental, 0.05 m, 0.1 m and 0.15 m are selected as the excitation displacement. The upper end of the force sensor is connected with the push rod of the excitation device, and the lower end is connected with the connecting rod of the simulation ore bin. The physical quantity measured by the sensor is converted into an electrical signal by the transmitter, and then the signal is transmitted to the upper computer through the RS-485 converter, the real-time force change curve is directly displayed in the upper computer. Rs-485 interface adopts differential signal transmission mode and does not need to detect signals relative to a certain reference point. The system only needs to detect the potential difference between two lines, therefore RS-485 converter is used here^36^. The simulated ore bin can be divided into cylindrical and rectangular shapes, as shown in Fig. 2c. The dimensions of cylindrical ore bin(diameter *D* × height *L*) are respectively $100{\text{mm}} \times 50{\text{mm}}$,$150{\text{mm}} \times 50{\text{mm}}$,$150{\text{mm}} \times 100{\text{mm}}$,$200{\text{mm}} \times 50{\text{mm}}$,$200{\text{mm}} \times 100{\text{mm}}$,$200{\text{mm}} \times 150{\text{mm}}$, and the rectangular ore bin (length × width × height) is $200{\text{mm}} \times 200{\text{mm}} \times 150{\text{mm}}$. There are altogether seven ore bin shapes to be measured, and the ore bin is located 1.5 m underwater.

## Results and discussion

Six groups of cylindrical and one group of rectangular ore bin are selected as the research objects, and the variation rules of resistance coefficient, force and acceleration under different length-diameter ratio, shape and additional mass are analyzed. The conclusions can provide reference data for the vibration control of the mining system in the next step.

## Research on resistance characteristics of simulated ore bin

**Figure 3:**
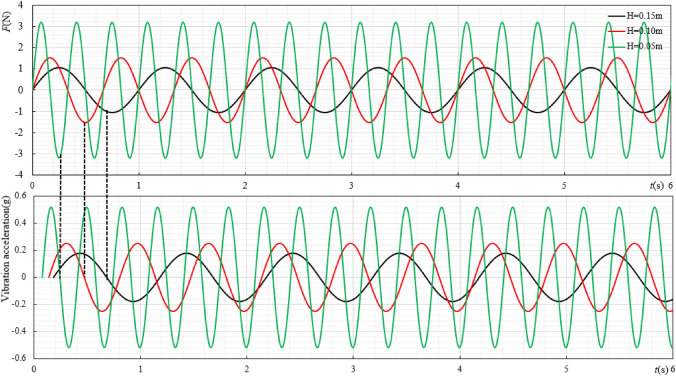
The size of the ore bin is $100{\text{mm}} \times 50{\text{mm}}$, the water depth is 1.5 m and $f_{0.05} = 3{\text{Hz}}$,$f_{0.1} = 1.5{\text{Hz}}$,$f_{0.15} = 1{\text{Hz}}$.The variation law of ore bin force and acceleration under different excitation displacements.

**Figure 4:**
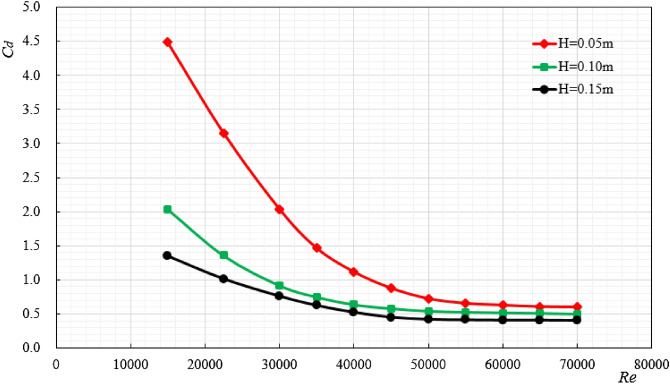
The variation law of resistance coefficient under different excitation displacements.

The cylindrical ore bin shape is selected as the research object, and the size is $100{\text{mm}} \times 50{\text{mm}}$. When the excitation displacement (*H*) is 0.05 m, 0.10 m and 0.15 m respectively, the change of force and acceleration during the ore bin movement is shown in Fig. 3, and the regular change between the resistance coefficient(*C*_*d*_) of the ore bin and Reynolds number(*Re*) is shown in Fig. 4.

When the acceleration is 0, only the relationship between the drag coefficient and the force can be considered. Figure 3 shows the changes of force and acceleration under different excitation displacements with *Re* = 15,000. It can be seen from Fig. 4 that when the acceleration is 0, the larger the excitation displacement is, the smaller the corresponding force value is. Figure 4 is the law change diagram of the ore bin resistance coefficient and Reynolds number. It can be seen from the figure that when the excitation displacement is constant, the resistance coefficient decreases with the increase of Reynolds number. This is because when the Reynolds number is small, the ore bin has a strong viscous effect and a larger force is needed to maintain the motion, so, the resistance coefficient is large at this time. As the Reynolds number increases, the viscosity effect on the ore bin weakens, so the resistance coefficient decreases. When the excitation displacement changes, increasing the excitation displacement will reduce the overall resistance coefficient of the ore bin. This is because when the excitation displacement increases, the excitation speed does not change, the excitation frequency becomes smaller, the effect of inertia force is weakened, the overall force of the system is reduced, and the resistance coefficient will show a downward trend. In this experimental study, when the Reynolds number is very large (up to 60,000), the resistance coefficient will remain stable and will not change with the increase of Reynolds number. When the excitation displacements are 0.05 m, 0.10 m and 0.15 m respectively, the drag coefficients are stable around 0.41, 0.50 and 0.60 with the increase of Reynolds number.

### The variation law of drag coefficient under different aspect ratio

**Figure 5:**
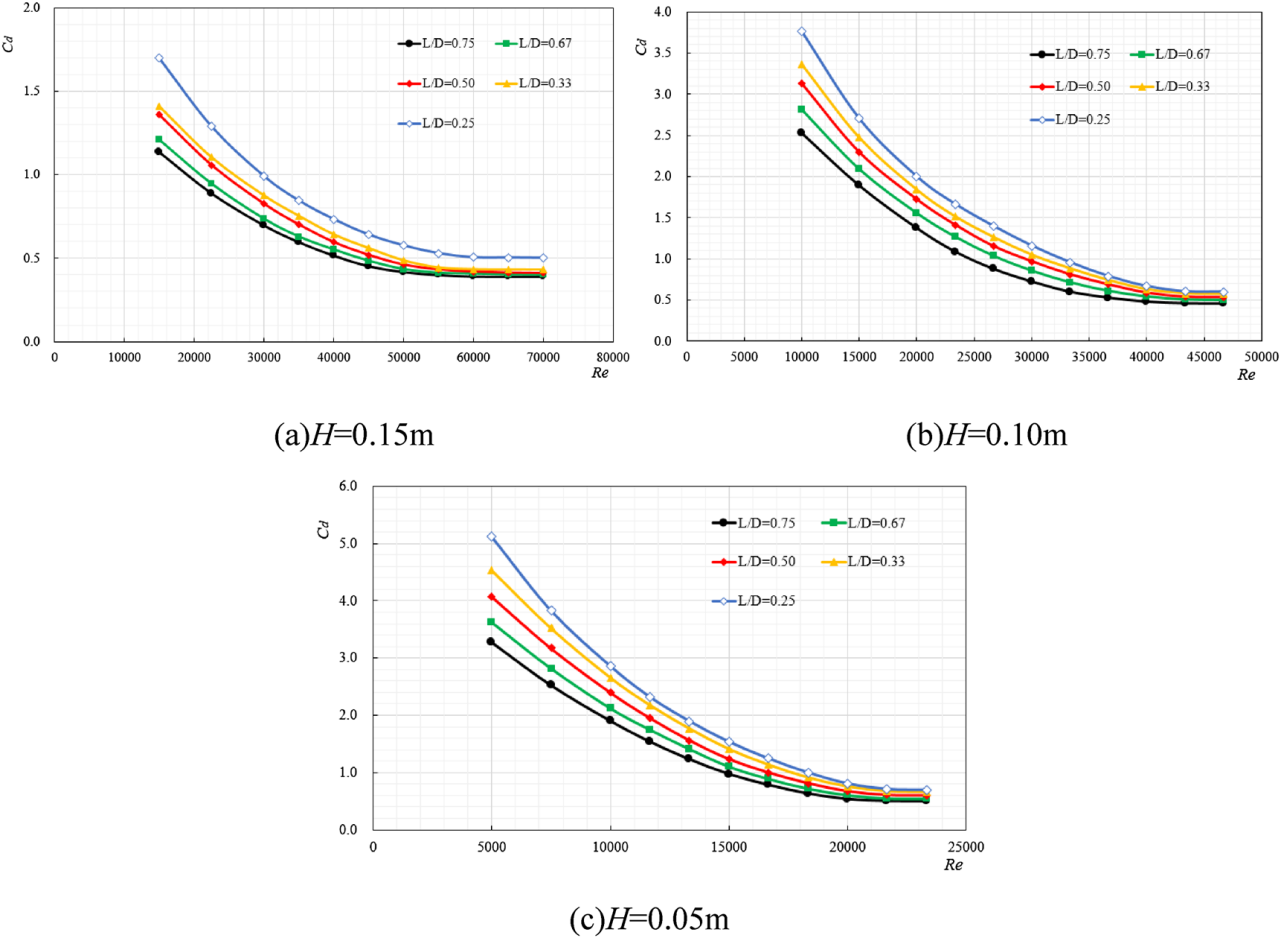
The variation law of resistance coefficient under different length-diameter ratios.

The other five ore bins were selected as research objects, and their sizes were $150{\text{mm}} \times 50{\text{mm}}$,$150{\text{mm}} \times 100{\text{mm}}$,$200{\text{mm}} \times 50{\text{mm}}$,$200{\text{mm}} \times 100{\text{mm}}$,$200{\text{mm}} \times 150{\text{mm}}$, the corresponding length-diameter ratios (*D*/*L*) were 0.33, 0.66, 0.25, 0.5 and 0.75. The regular changes between the ore bin resistance coefficient and Reynolds number under the same excitation displacement with different length-diameter ratios are shown in Fig. 5.

**Figure 6:**
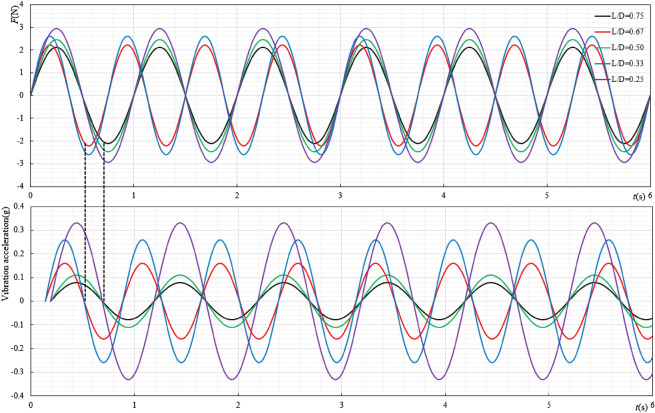
$f_{0.25} = f_{0.5} = f_{0.75} = 1{\text{Hz}}$, $f_{0.33} = f_{0.67} = 1.33{\text{Hz}}$, the variation diagram of force and acceleration under different length-diameter ratios.

It can be seen from Fig. 5a–c that changing the size of the ore bin will not change the original variation law of the resistance coefficient, that is, the resistance coefficient gradually decreases with the increase of Reynolds number and stabilizes within a certain range, reducing the displacement of excitation will increase the resistance coefficient of the ore bin. Under the same excitation displacement, the smaller the length-diameter ratio is, the larger the resistance coefficient is. This is because it can be seen from Fig. 6 that the smaller the length-diameter ratio is, the larger the force generated by the system is, and the larger the corresponding resistance coefficient is. Similarly, when the Reynolds number increases to a certain extent, the resistance coefficient will decrease to a stable value. The stable value under different length-diameter ratios is shown in Table 1.

**Table 1:** Stable value of drag coefficient under different length-diameter ratios.

*L*/*D*	*C*_*d*_ (*H* = 0.15 m)	*C*_*d*_ (*H* = 0.10 m)	*C*_*d*_ (*H* = 0.05 m)
0.75	0.39	0.45	0.50
0.67	0.40	0.49	0.55
0.50	0.41	0.53	0.60
0.33	0.43	0.57	0.66
0.25	0.50	0.60	0.69

### The variation law of drag coefficient under different external shape

**Figure 7:**
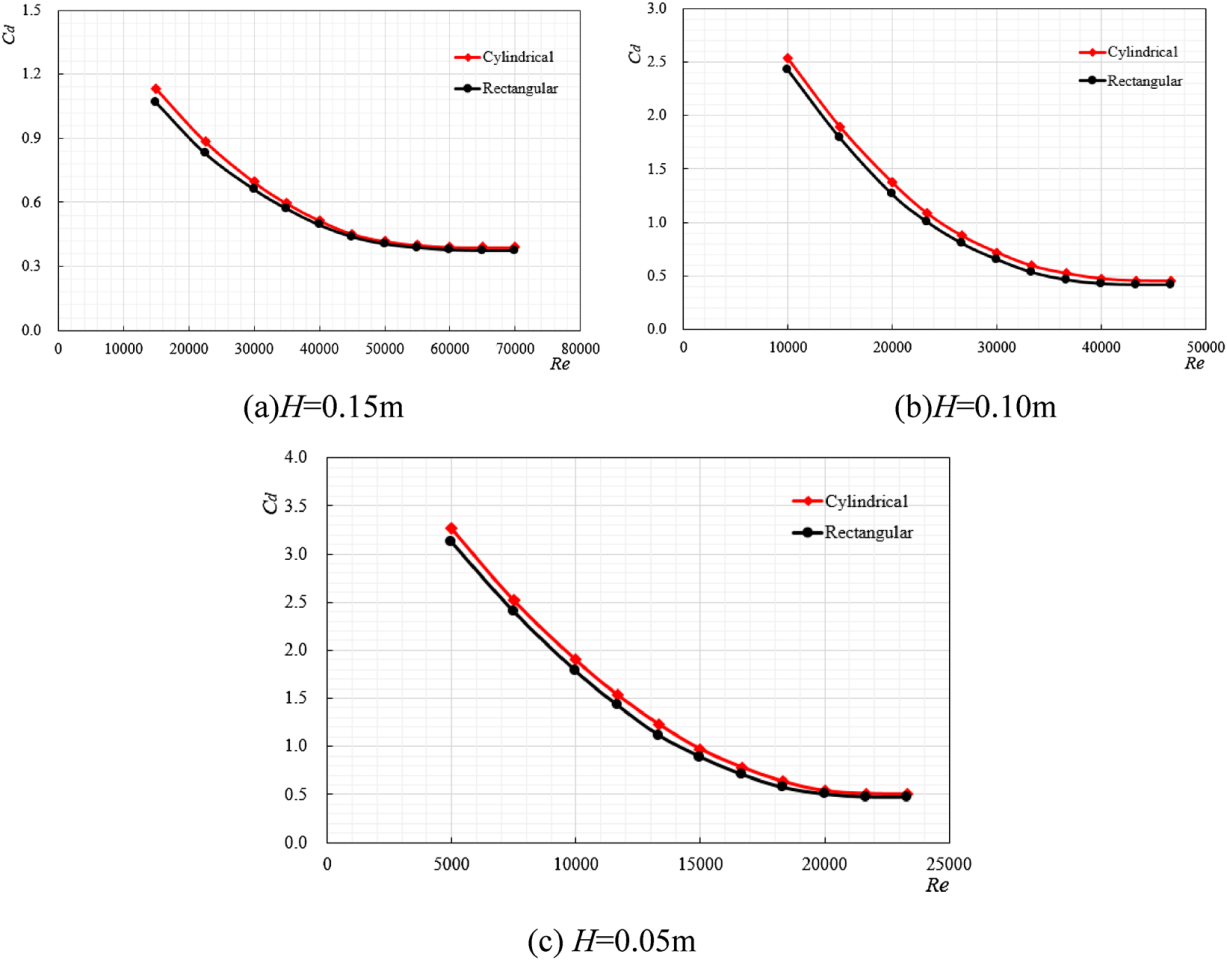
The resistance coefficient variation law diagram under different ore bin shape.

Cylindrical (200 × 150 mm) and rectangular (200 × 200 × 150 mm) ore bins were selected as the research objects, with the same characteristic length and different external shapes, and the length-diameter ratio is 0.75. When the excitation displacement is 0.05 m, 0.10 m and 0.15 m respectively, the regular changes between the ore bin resistance coefficient and Reynolds number is shown in Fig. 7.

**Figure 8:**
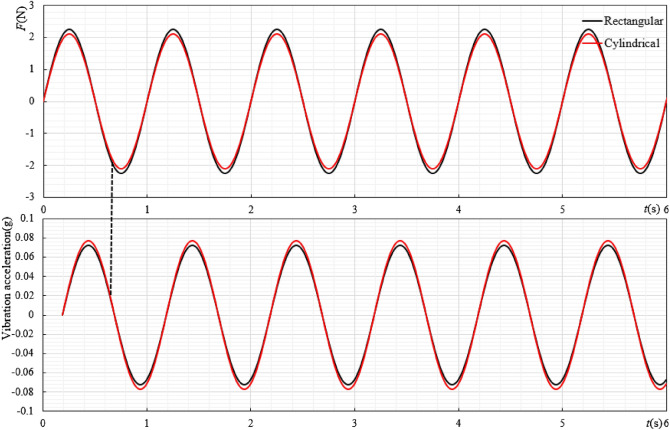
$H = 0.15{\text{m}}$,$f = 1{\text{Hz}}$, the water depth is 1.5 m, the variation law diagram of force and acceleration under different ore bin shape.

As can be seen from Fig. 7, when the characteristic length of the ore bin is the same and the external shape is different, its original law does not change. No matter the shape is rectangular or cylindrical, the resistance coefficient decreases with the increase of Reynolds number and finally stabilizes around a certain range. It can be seen from Fig. 8 that when the characteristic length of the ore bin is the same, the corresponding force value under the condition of 0 m/s^2^ does not change much, but the force area of different shapes of ore bin is different. The rectangular shape is slightly larger than the cylindrical shape, so the resistance coefficient of the cylindrical shape is larger than that of the rectangular shape. In the actual working conditions, the influence of ore bin on longitudinal vibration and transverse towing of the mining pipe should be considered simultaneously. For the longitudinal vibration, the large resistance coefficient can generate the large movement resistance, consume the longitudinal excitation force, and reduce the vibration amplitude. In the process of transverse towing, the shape of the side of the ore bin is streamlined, which can reduce the force of the mining pipe during towing and shorten the distance of the transverse deviation. Therefore, the cylindrical shape is selected as the external shape of the ore bin.

### The variation law of drag coefficient under different additional weight

**Figure 9:**
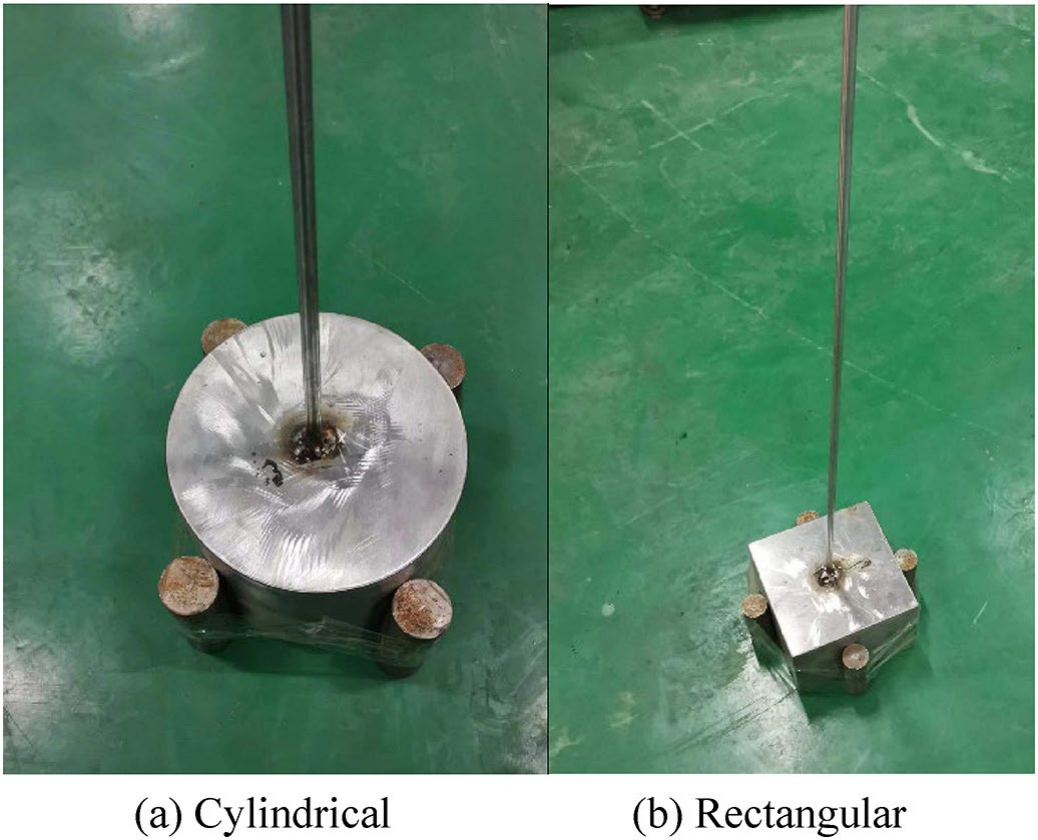
Simulated ore bin with different weight block diagrams.

**Figure 10:**
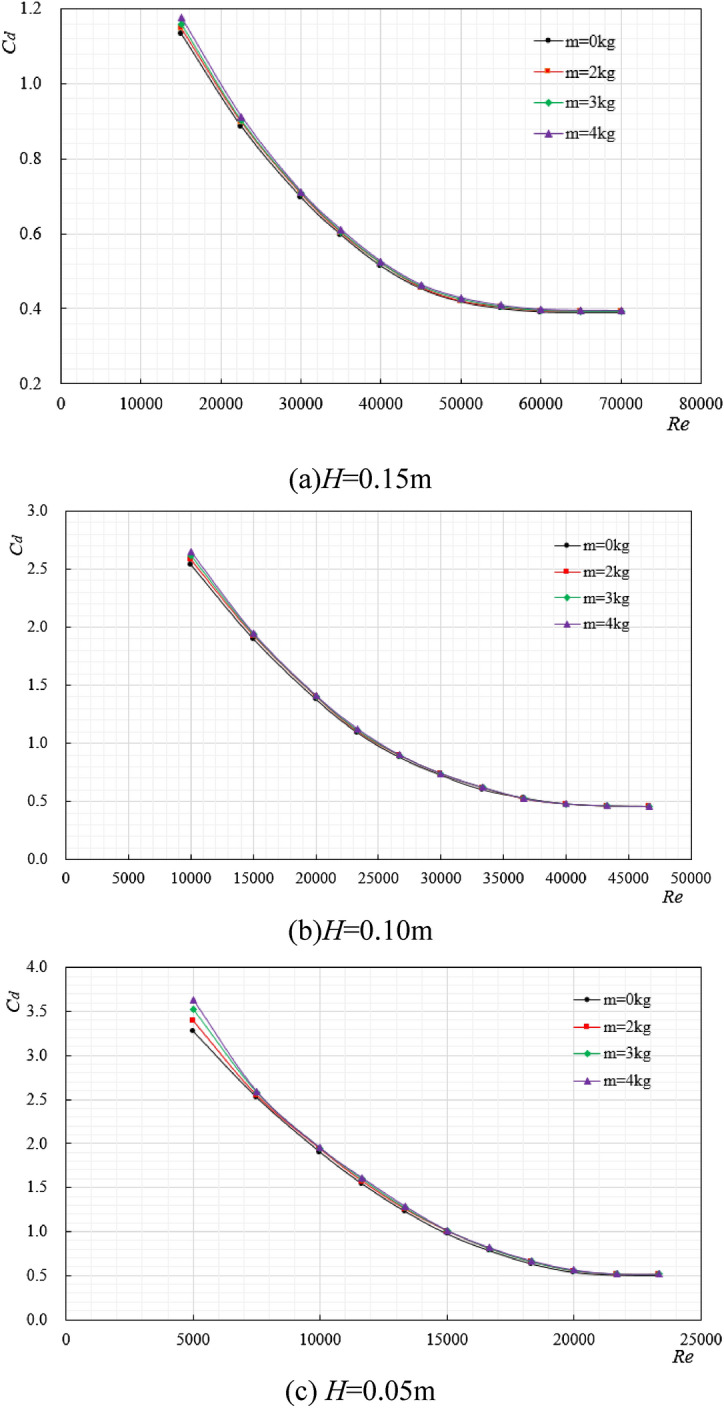
The variation law diagram of resistance coefficient of cylindrical ore bin under different additional weights.

**Figure 11:**
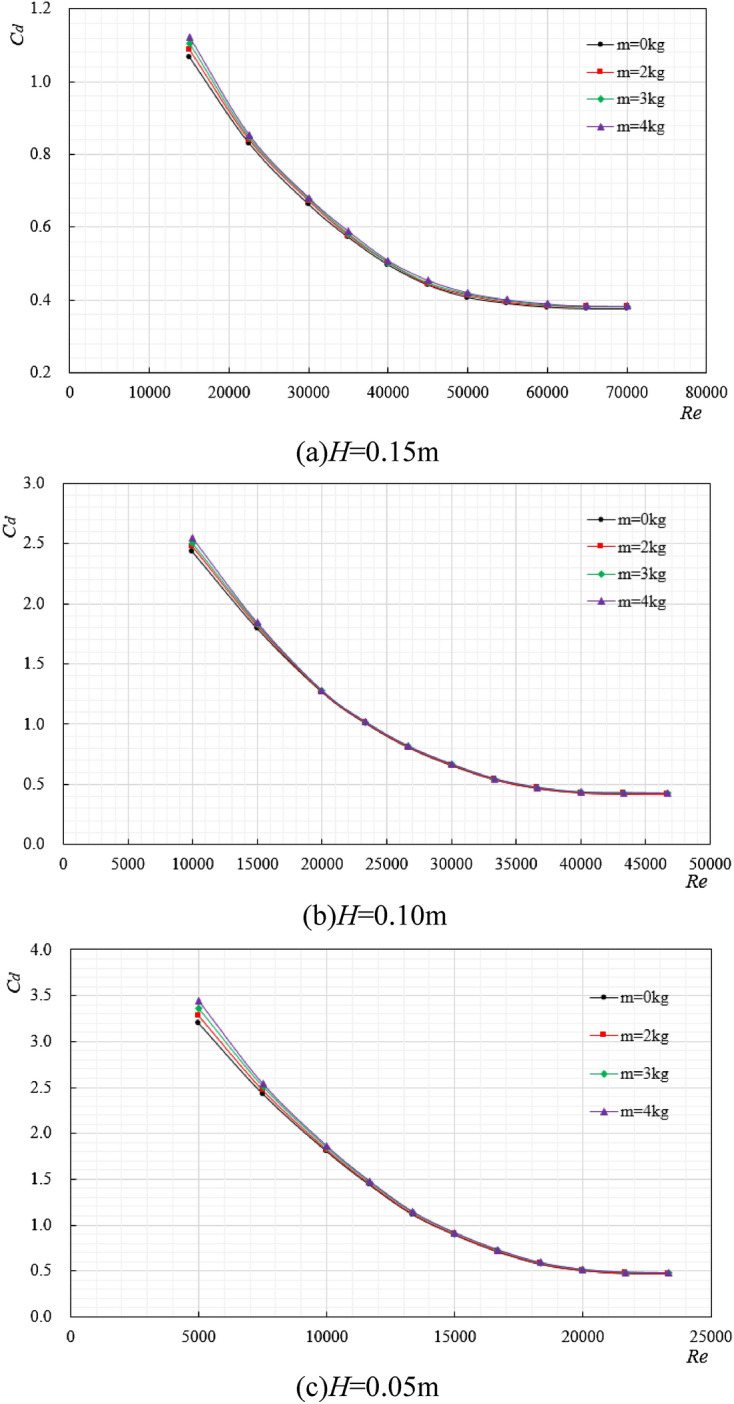
The variation law diagram of resistance coefficient of rectangular ore bin under different additional weights.

Cylindrical (200 × 150 mm) and rectangular (200 × 200 × 150 mm) ore bins were selected as the research objects, with the same characteristic length and different external shapes, and the length-diameter ratio is 0.75. When the ore bin was attached with objects of different weights (as shown in Fig. 9), the regular changes between the ore bin resistance coefficient and Reynolds number are shown in Figs. 10 and 11.

As can be seen from Fig. 9, weight blocks are attached to the side of the simulated ore bin, each weight block is 1 kg, and 2, 3, 4 weight blocks are successively added. The variation law of resistance coefficient under different additional weight was studied. It can be seen from Figs. 10 and 11 that no matter the ore bin is cylindrical or rectangular, the increase of additional weight does not change the change law of resistance coefficient. At the initial stage of Reynolds number, the added weight has a great influence on the resistance coefficient, and the increase of the added weight will increase the drag coefficient. However, at high Reynolds number, the drag coefficient will become stable and its value will not change with the increase of the added weight. Tables 2, 3, 4 shows the stable values of the resistance coefficient at high Reynolds number under different excitation displacements, indicating that as long as the external shape of the ore bin is determined, the resistance coefficient will not change significantly at high Reynolds number, while the stable value of the cylindrical resistance coefficient is large at high Reynolds number under the same excitation displacements. In the field production of mining system, the ore bin is a transfer station for the collection and transmission of seabed resources, and the additional weight is constantly changing. The research on the relationship between the additional weight and the resistance coefficient has an important guiding role in the actual working conditions.

**Table 2:** *H* = 0.15 m, stable value of resistance coefficient.

Weight block	*m* = 0 kg	*m* = 2 kg	*m* = 3 kg	*m* = 4 kg
*C*_*d*_ (Cylindrical)	0.390	0.391	0.391	0.392
*C*_*d*_ (Rectangular)	0.378	0.380	0.381	0.381

**Table 3:** *H* = 0.10 m, stable value of resistance coefficient.

Weight block	*m* = 0 kg	*m* = 2 kg	*m* = 3 kg	*m* = 4 kg
*C*_*d*_ (Cylindrical)	0.454	0.456	0.457	0.457
*C*_*d*_ (Rectangular)	0.420	0.422	0.422	0.423

**Table 4:** *H* = 0.05 m, stable value of resistance coefficient.

Weight block	*m* = 0 kg	*m* = 2 kg	*m* = 3 kg	*m* = 4 kg
*C*_*d*_ (Cylindrical)	0.502	0.505	0.508	0.510
*C*_*d*_ (Rectangular)	0.470	0.471	0.474	0.475

To sum up, based on the assumptions and theories in Sect. 2, the built test device for testing the longitudinal resistance coefficient can better measure the variation law of the resistance coefficient under different aspect ratios, shapes and additional weights, which verifies the rationality of the hypothesis and theoretical application, but the next step is to research the change law of inertia force coefficient in order to get better results.

## Conclusions

In this paper, the longitudinal resistance coefficient of simulated ore bin is studied. A experimental system was set up to test the longitudinal resistance coefficient, and the variation law of the resistance coefficient with Reynolds number under different length-diameter ratio, shape and additional weight of the ore bin was analyzed. The conclusion can be helpful for the selection of the actual ore bin and the vibration reduction design of the whole mining system. Determine the resistance coefficient value of ore bin under different working conditions, for the next step to research the influence of ore bin on pipeline vibration, determine the optimal shape of ore bin in practical work to lay a foundation. The main conclusions are as follows:

1. When the excitation displacement is unchanged, the resistance coefficient of the ore bin decreases gradually with the increase of Reynolds number and finally stabilizes near a certain value. Under the same size of the ore bin, reducing the excitation displacement of the system will increase the overall resistance coefficient of the ore bin.
2. When the excitation displacement is constant, increasing the length-diameter ratio of the ore bin can reduce the overall resistance coefficient, but changing the length-diameter ratio will not change the law that the resistance coefficient decreases gradually with the increase of Reynolds number, and the resistance coefficient is the same under the same length-diameter ratio.
3. When the characteristic length and excitation displacement are the same, the resistance coefficient of the cylindrical shape is greater than that of the rectangular shape, the design of the ore bin shape should not only meet the requirements of reducing the longitudinal vibration, but also meet the standard of keeping the offset distance as small as possible in the transverse towing, so the shape of the ore bin in the actual design and production is designed to be cylindrical.
4. For the same shape of the ore bin, changing the added weight at a high Reynolds number (Re ≥ 20,000) will not change the resistance coefficient of the ore bin, while increasing the added weight at a low Reynolds number will increase the resistance coefficient.

## Data Availability

The data used to support the findings of this study are available from the corresponding author upon request.
